# Effect of CeO_2_ on Impact Toughness and Corrosion Resistance of WC Reinforced Al-Based Coating by Laser Cladding

**DOI:** 10.3390/ma12182901

**Published:** 2019-09-08

**Authors:** Weizhan Wang, Zhigang Chen, Shunshan Feng

**Affiliations:** 1 College of Mechatronic Engineering, North University of China, Taiyuan 030051, China; 2 School of Mechatronical Engineering, Beijing Institute of Technology, Beijing 100081, China

**Keywords:** laser cladding, CeO_2_, S420 steel, impact toughness, corrosion resistance

## Abstract

WC reinforced Al-based coating with added CeO_2_ was prepared on the surface of S420 steel by laser cladding. The microstructure and structure of the coatings were analyzed by scanning electron microscope, X-ray diffractometer and optical profiler. The mechanical properties and corrosion properties of the coatings were studied by microhardness tester, friction and wear tester, Charpy impact tester, and electrochemical workstation. The results show that the coating is mainly composed of Al-phase, continuous-phase, and hard reinforced-phase WC, and the coating and substrate show good metallurgical bonding. When the content of CeO_2_ is 1%, the fine grain strengthening effect is obvious, and the impact toughness of the coating is obviously improved. Appropriate amount of rare earth CeO_2_ can significantly improve the hardness of the coating. When the content of CeO_2_ is more than 1%, the wear resistance of the coating decreases. The coating prepared with different CeO_2_ content has higher impedance and corrosion resistance than that of the substrate. At 1% CeO_2_ content, the coating has the best corrosion resistance.

## 1. Introduction

Offshore platform is a tool for human beings to develop marine resources, which is used in severe marine working environments such as waves, tide, storm and cold current ice. These environments determine that offshore platform steel must have high strength, toughness, corrosion resistance, fatigue resistance, and layered tear resistance [1,2,3]. At present, S420 steel has been widely used in the construction of offshore oil platform. As national offshore oil and gas development is gradually moving towards the deep water area, the thickness, quality, and construction difficulty of the plate used in offshore engineering structural steel are increasing [4,5,6,7]. Simultaneously, the marine deep water area environment is complex, and usual iron and steel materials have been unable to meet the needs of use [8,9]. Laser cladding ceramic particle reinforced metal-based composite coating has the advantages of high strength, high hardness, high wear resistance, and high corrosion resistance, which is an effective way to solve the failure of materials in complex environment [10,11,12]. For example, Ye et al. have prepared V_8_C_7_ reinforced Fe based composite coatings with different volume fraction. With the increase of volume fraction of reinforced-phase, the hardness of the composite coating increased, while the impact toughness decreased from 8.1 J/cm^2^ to 4.7 J/cm^2^. When the volume fraction of the reinforced phase is less than 24%, the wear resistance of the coating increases with the increase of V_8_C_7_ content, and when the volume fraction is more than 24%, the wear resistance decreases due to the crushing of particles and the generation of microcracks [13].

There are great differences in physical and chemical properties between ceramic particles and matrix materials, such as thermal expansion coefficient, interfacial strength, brittleness of reaction products, etc., which may lead to the plasticity and toughness of the composite coating lower than that of the matrix material. Under the condition of impact load. Composites are prone to fracture and early failure, thus decreasing corrosion resistance in the marine environment, which greatly limits the application and development of matrix materials [14,15]. Rare earth elements can improve the microstructure and properties of laser cladding coating, increase the compactness of coating structure, reduce the coefficient of thermal expansion between coating and substrate, and reduce the thermal stress, impurities, and cracks in the coating [16]. Zhang et al. studied the effect of CeO_2_ on the microstructure and corrosion resistance of TiC-VC reinforced Fe-based laser cladding coating. With increasing CeO_2_ addition, the number of flake pearlite increased, the amount of retained austenite decreased, and the corrosion resistance of cladding coating increased at first and then decreased. When 0.5 wt.% CeO_2_ is added, the corrosion resistance of the coating is the best [17]. Sun et al. prepared (Ti, Nb)C/Ni coatings with different amount of CeO_2_. The results showed that the size of (Ti, Nb)C particles was suppressed by rare earth element Ce, which is prone to accumulate at the interface of grain boundary or crystalline phases. The unmelted CeO_2_ can also be used as the basis for heterogeneous nucleation of (Ti,Nb)C, Cr_23_C_6_, and Cr_7_C_3_ particles. In addition, with increasing CeO_2_ content, the tensile properties of the coating were improved, and the fracture behavior changed from quasi-cleavage fracture to brittle fracture [18].

From the literature review, the ceramic particle reinforced metal-based composite coating can significantly improve the hardness of the coating and improve the corrosion resistance to a certain extent. However, with the increase of the volume fraction of ceramic particles, the impact toughness of the composite coating decreases seriously, resulting in particle breakage or matrix cracking in some environments, and the corrosion resistance decreases instead. Therefore, improving the impact toughness of composite coating without losing their corrosion resistance has become the research focus of particle reinforced metal-based materials [19,20]. 

In this work, we mainly use the idea of rare earth purification to prepare particle reinforced metal-based composite coatings with different rare earth content. The microstructure, impact toughness and corrosion resistance of the composite coating were studied systematically by scanning electron microscope, X-ray diffractometer, Charpy impact tester, and electrochemical workstation, and the action mechanism of rare earth was analyzed. It provides an experimental basis for the effective combination of impact toughness and corrosion resistance of ceramic particles reinforced metal-based composites.

## 2. Experimental

**Figure 1:**
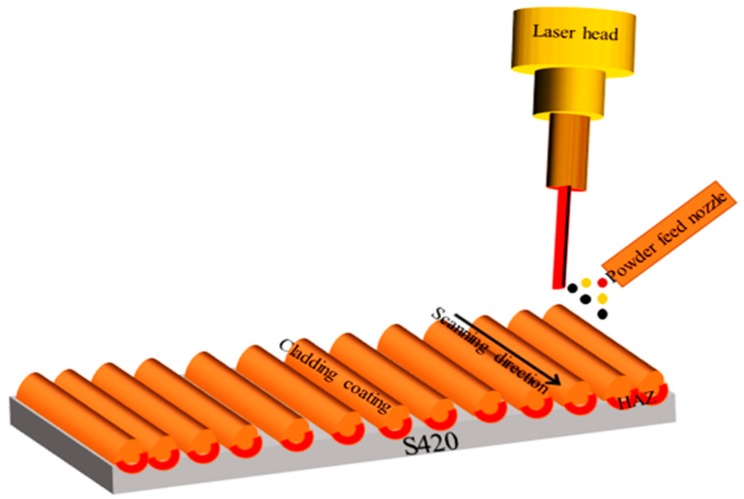
Schematic of laser cladding process.

The experimental material was European standard S420 structural steel (HBIS WU Steel, Shijiazhuang, China). Table 1 shows the chemical composition of S420 steel. The size of the matrix material is 60 mm × 30 mm × 3 mm. The surface is mechanically polished and washed repeatedly with acetone. The cladding powder material is Al powder, Ni powder, and WC powder, which are mixed according to the mass ratio of 6:1:2, the particle size of the mixed powder is 45–100 μm. Rare earth CeO_2_ was added at the mass ratio of 0%, 1%, 1.5%, and 2%, respectively. It was fully mixed and ground by ball mill, and then put into the drying box at 60 °C for 30 min. Each group of powder samples was tested by laser cladding three times. According to the macroscopic test results of the three cladding samples, the samples with better cladding quality were selected to test the microstructure and properties. The laser cladding test is completed by YSL-4000 optical fiber laser processing system using synchronous powder-feeding cladding. Figure 1 shows the principle of laser cladding process. The laser power is 1300 W, the scanning speed is 0.5 m/min, the powder feeding rate is 8 g/min, and the spot diameter is 3 mm. The laser cladding adopts the method of multichannel and multilayer lap, and the lap rate is 40%. After the cladding is completed, the sample is cut into the size of 10 mm × 10 mm × 3 mm by wire cutting. After the completion of the cladding experiment, the surface of the cladding coating was polished step by step with water-scrubbed paper, and the finished sample was mechanically polished with Al_2_O_3_ polishing solution and de-oiled by acetone ultrasonic.

**Figure 2:**
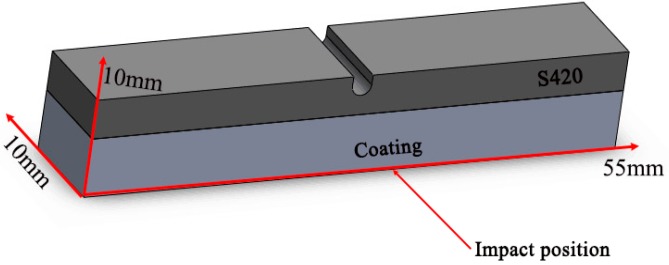
Schematic diagram of impact specimens.

The microstructure and element composition of the coating were characterized by JSM-6510 scanning electron microscope (SEM, JEOL, Tokyo, Japan) and energy-dispersive spectroscopy (EDS, JEOL, Tokyo, Japan), and the phase composition of the coating was analyzed by X-ray diffractometer (XRD, Rigaku, Tokyo, Japan). The hardness of the sample was measured by 430SVD Vickers hardness tester (PROCEQ, Schwerzenbach, Switzerland). The test method was parallel to the horizontal section direction, the loading load was 200 g, the loading time was 15 s, a point was hit every 50 μm from the surface of the cladding layer to the substrate, and the average value was measured three times at the same depth level. The wear properties of the coating were tested by reciprocating sliding friction and wear tester (Chinese Academy of Sciences, China). The test conditions and technological parameters of wear are as follows, steel ball (grinding material), air (test medium), loading load of 250 g, wear scar radius of 4 mm, running time of 30 min. After the test was completed, the wear weight loss was measured by BT25S electronic analysis balance (Sartorius, Goettingen, Germany), and the wear scar morphology was observed by scanning electron microscope. The Charpy U-shaped notched impact specimen is cut parallel to the laser scanning direction along the cladding layer, and the sample size is 10 mm × 10 mm × 55 mm, which ensures that all the impact toughness specimens are in the cladding region, as shown in Figure 2. The impact test was carried out with JXB-300 pendulum impact tester. After the experiment was completed, the impact fracture was observed and analyzed by VHX-1000C optical profiler (KEYENCE, Osaka, Japan) and scanning electron microscope (SEM). The corrosion resistance of the coating was evaluated by CS350 electrochemical workstation (Corrtest, Wuhan, China). The test medium is a 3.5% NaCl solution, the electrochemical workstation uses a three-electrode system, the working electrode is sample, the reference electrode is saturated calomel electrode, and the auxiliary electrode is platinum electrode. The potentiodynamic scanning rate is 1 mV/s, the sampling frequency is 0.5 Hz, the measuring potential range is −1 to 1 V, and the test time is 1800 s. The frequency range measured by EIS (Electrochemical Impedance Spectroscopy) is 10^−1^ to 10^5^ and the test time is 300 s. After the test is completed, the data will be fitted by ZSimDemo software.

## 3. Results and Discussion

### 3.1. Microstructures Analysis

**Figure 3:**
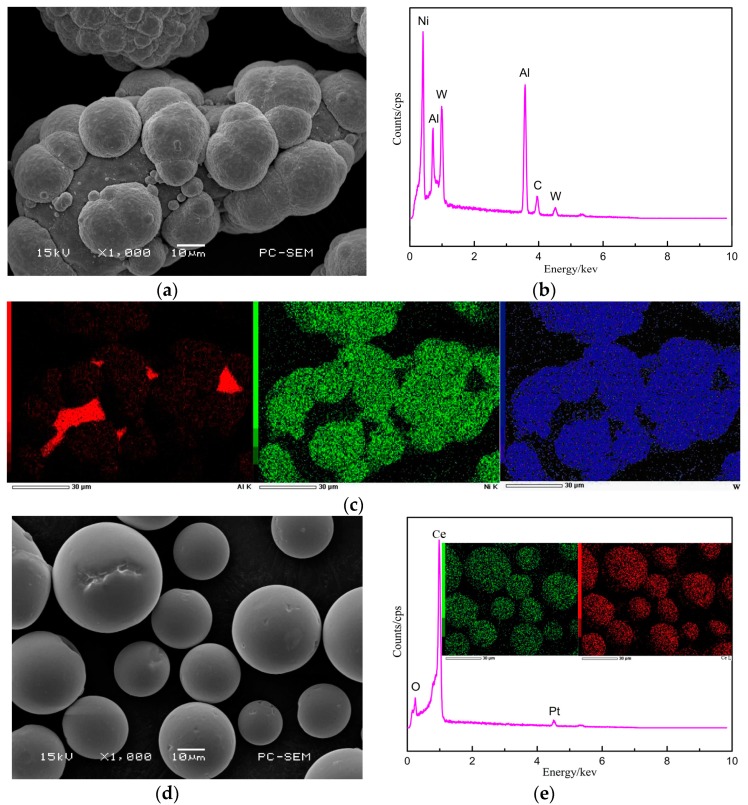
Morphologies (**a**,**d**) and energy-dispersive spectroscopy (EDS) analysis (**b**,**c**,**e**) of powders: (**a**) Ni/Al/WC mixed powder and (**d**) CeO_2_ powder.

Figure 3 shows the morphology and energy spectrum analysis of the powder used in cladding. The particle size of Al powder is about 80 μm and the morphology is irregular. The morphology of WC powder is regular, WC powder distributed on the surface of Al powder, and the particle size is about 20 μm. After ball-milling, the mixed powder does not produce impurity elements. The morphology of CeO_2_ powder is regular spherical, the particle size is 15 to 30 μm, and the element distribution is uniform.

**Figure 4:**
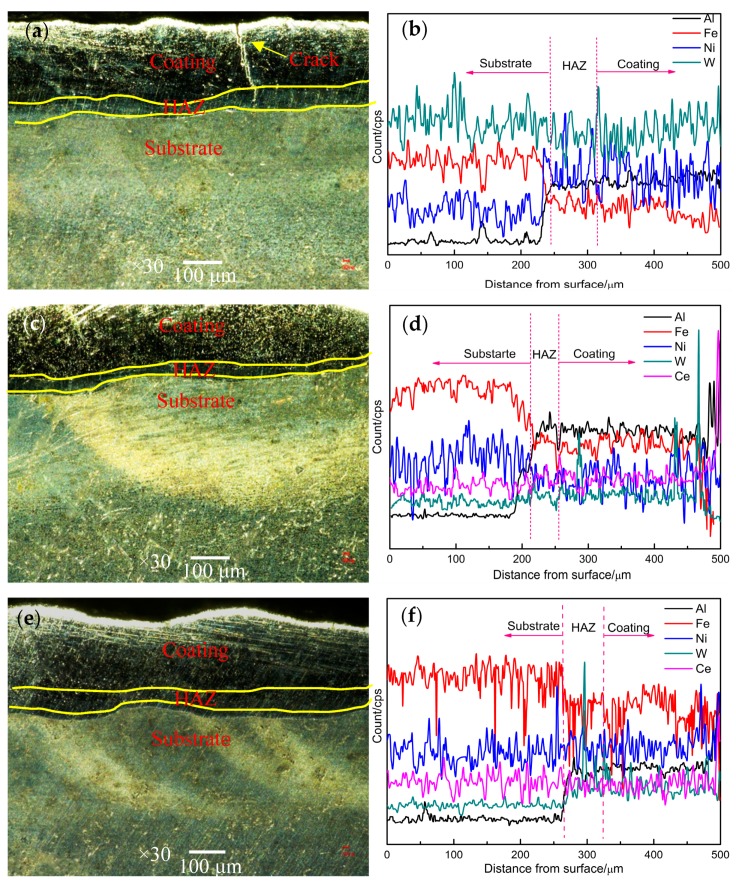

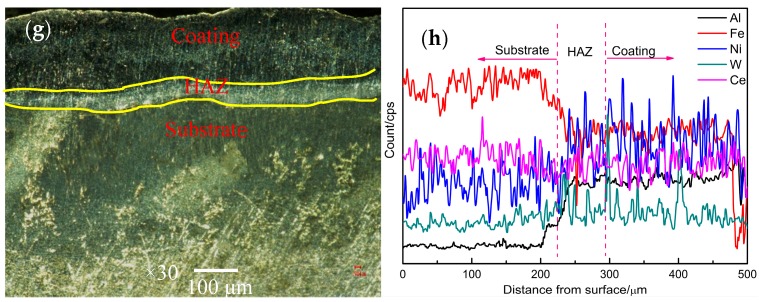
Cross section morphology (**a**,**c**,**e**,**g**) and linear scanning diagram (**b**,**d**,**f**,**h**) of coatings with different CeO_2_ content: (**a**,**b**) 0.0% CeO_2_, (**c**,**d**) 1.0% CeO_2_, (**e**,**f**) 1.5% CeO_2_, and (**g**,**h**) 2.0% CeO_2_.

Figure 4 shows the cross section morphology of the cladding coating prepared with different CeO_2_ content. When the rare earth CeO_2_ is not added, an obvious crack appears in the cross section of the coating, and the crack gradually extends to the heat affected zone (HAZ). The elements in the coating region are mainly composed of Al, Fe, Ni, and W, and the distribution of Ni and W elements has great fluctuations. The appearance of Fe elements shows that the Fe in the substrate diffuses into the coating through the molten pool, as a result, the interface between the substrate and the coating is metallurgical bonded [21]. The depth of HAZ is 70 μm and the thickness of coating is 700 μm. According to the formula of dilution rate, it can be concluded that the dilution rate of coating is 9% without adding rare earth. When the rare earth content is 1%, the cross section of the coating is smooth and there are no macroscopic defects. The main elements in the coating are Al, Fe, Ni, W, and Ce. The depth of HAZ is 50 μm and the dilution rate is 6.6%, the bonding zone between the coating and the substrate forms a reasonable composition gradient, and the microstructure of the coating tends to be homogenized. When the content of CeO_2_ increased to 1.5%, pores began to appear in the coating, but the number of pores was less. The distribution of W element in the coating fluctuates obviously, the depth of HAZ is expanded to ~60 μm, and the dilution rate is gradually increased to 7.8%. When the rare earth content increases to 2%, the cross section of the coating is rough, the distribution of Ni and W elements is uneven, and the dilution rate increases to 8.4%.

**Figure 5:**
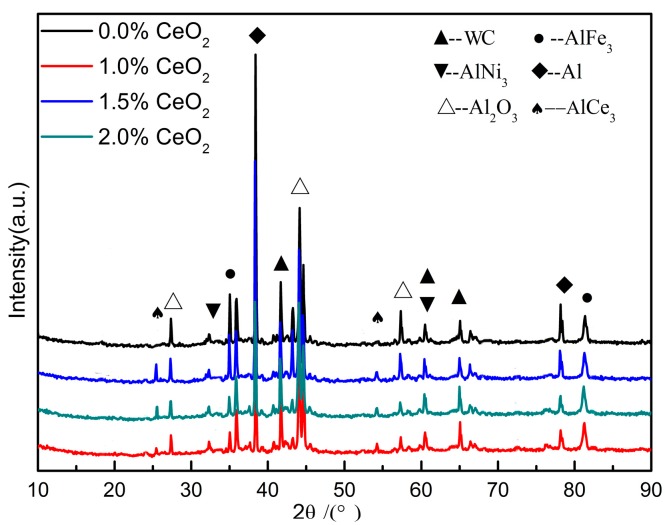
XRD patterns of coatings with different CeO_2_ content.

The prepared coating was characterized by XRD, and the XRD pattern is shown in Figure 5. It can be seen that the coating is mainly composed of matrix Al phase, continuous-phase, and hard reinforced-phase WC. The impurity phase of the coating with rare earth addition is obviously less than that of the coating without rare earth, and the crystal diffraction peaks of AlCe_3_ phase appear at approximately 25° and 55°, which indicates that rare earth CeO_2_ decomposes and reacts with Al at high temperature in order to purify the grain [22]. The appearance of AlFe_3_ phase shows that the Fe element in the substrate diffuses into the coating, resulting in the metallurgical bonding between the substrate and the coating [23]. In addition, the diffraction peak intensity of Al_2_O_3_ phase of the coating without rare earth addition is higher than that of other rare earth coatings, which indicates that rare earth has a certain oxidation resistance of the coating. The AlNi_3_ peak appears in all the four coatings. According to the calculated results of Gibbs free energy, the temperature of laser cladding is higher than 1000 K, and the AlNi_3_ phase is relatively stable.

**Figure 6:**
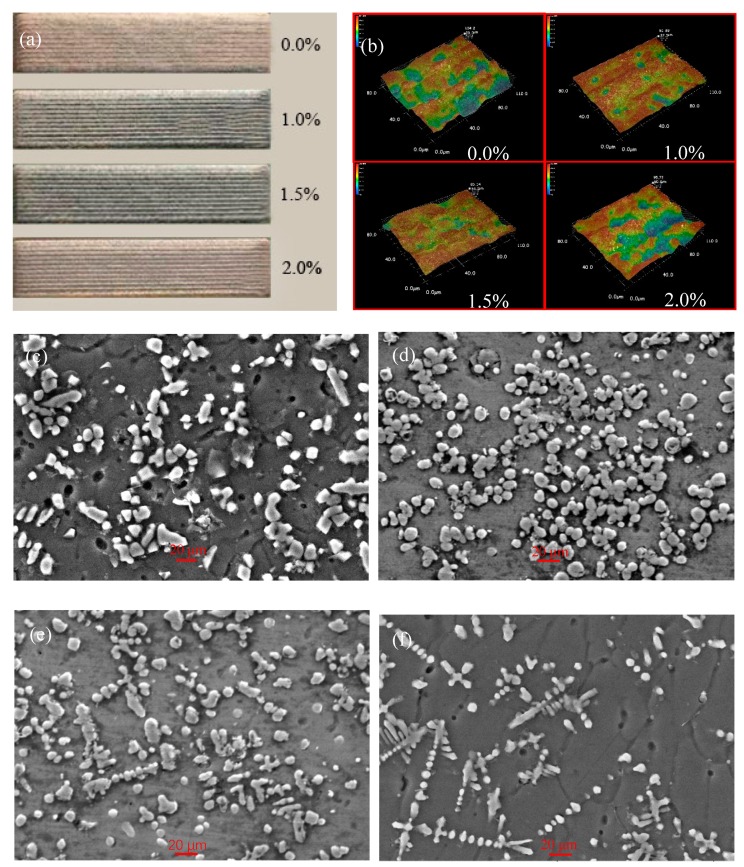
Macro-morphology (**a**), three-dimensional morphology (**b**), and micromorphology (**c**,**d**,**e**,**f**) of coatings with different CeO_2_ content: (**c**) 0.0% CeO_2_, (**d**) 1.0% CeO_2_, (**e**) 1.5% CeO_2_, and (**f**) 2.0% CeO_2_.

Figure 6 shows the macro-morphology, surface three-dimensional morphology and micromorphology of the coating. From Figure 6a, it can be seen that after the addition of rare earth CeO_2_, the surface of the coating is flat, the color is darker, the surface has metallic luster, and there are no macroscopic defects. From the perspective of three-dimensional morphology and microscopic morphology, when the rare earth CeO_2_ is not added, the surface of the coating is uneven, the roughness is large, and the surface shows undulating ripples. The grains are coarse and bonded to each other, showing rod shape and block shape [24]. When the content of CeO_2_ is 1%, the surface flatness of the coating is higher than that of the coating without rare earth addition, and the grains in the coating are obviously refined, showing granular shape and uniform distribution. This is mainly due to the fact that the addition of a small amount of rare earth can reduce the melting temperature, shorten the melting time, improve the flowability of the molten pool and facilitate the homogenization of the elements. When the content of CeO_2_ is 1.5%, it can be seen that the surface of the coating is relatively flat, but there are fewer pores and the microstructure of the coating is granular and flake. When the CeO_2_ content is further increased, the wavy pattern appears on the surface of the coating, the surface becomes uneven, the pores increase, and the tissue distribution is uneven. Therefore, the appropriate amount of rare earth can improve the quality of the cladding layer and refine the grain, but when the content of rare earth CeO_2_ is more than 1.5%, the quality of the coating begins to decline. Because of the strong electronegativity of rare earth, it shows strong chemical properties when the content is low, which can fill the defects of alloy phase in the molten pool, reduce the surface tension, promote the effective number of nuclei, and restrain the crystallization process, which leads to microstructure refinement. When the rare earth is excessive, the rare earth elements will dissolve in some metal compounds, resulting in less segregation at the grain boundary, reducing the undercooling at the grain boundary, promoting the activity of the grain boundary, and making the microstructure of the coating larger and the quality of cladding lower.

### 3.2. Properties Analysis

**Figure 7:**
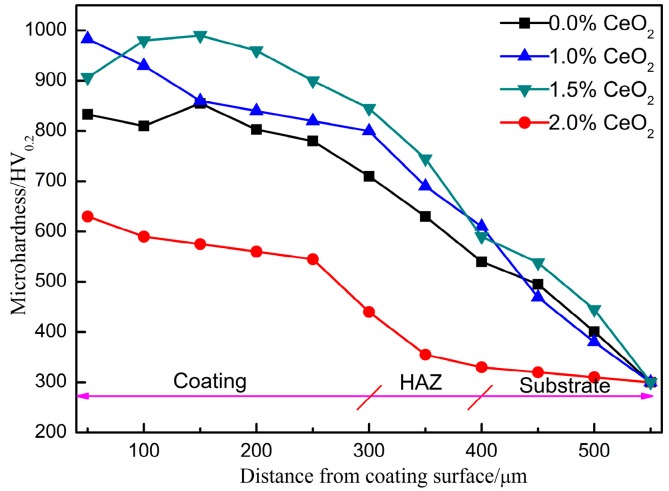
Microhardness distribution of coatings with different CeO_2_ content.

The microhardness distribution of the cladding coating is shown in Figure 7. According to the graph, the hardness of the four different coatings has the same trend, and the hardness decreases gradually from the coating surface to the substrate, especially in the heat-affected zone. In the bonding area between the substrate and the coating, the hardness of the coating gradually becomes stable. When the content of CeO_2_ is 1.0%, the maximum surface hardness of the coating is 983.3 HV_0.2_, which is nearly twice as much as that of the substrate. When the CeO_2_ content increases to 1.5%, the surface hardness of the coating decreases by 8%, and when the CeO_2_ content increases to 2.0%, the hardness of the coating decreases by 35%. It can be seen that the addition of appropriate amount of rare earth CeO_2_ can significantly improve the hardness of the cladding layer. When the content of CeO_2_ is more than 1%, the hardness increase trend is not obvious. On the one hand, because the strength of the metal is negatively related to the grain size, the smaller the grain size, the greater the strength of the metal and the higher the hardness of the material; on the other hand, in the nondynamic equilibrium process of laser cladding, the addition of rare earth elements can play the role of solid solution strengthening, which distorts the lattice size and promotes the improvement of hardness. Therefore, when the content of rare earth CeO_2_ is 1.0%, the hardness of the coating is the best.

**Figure 8:**
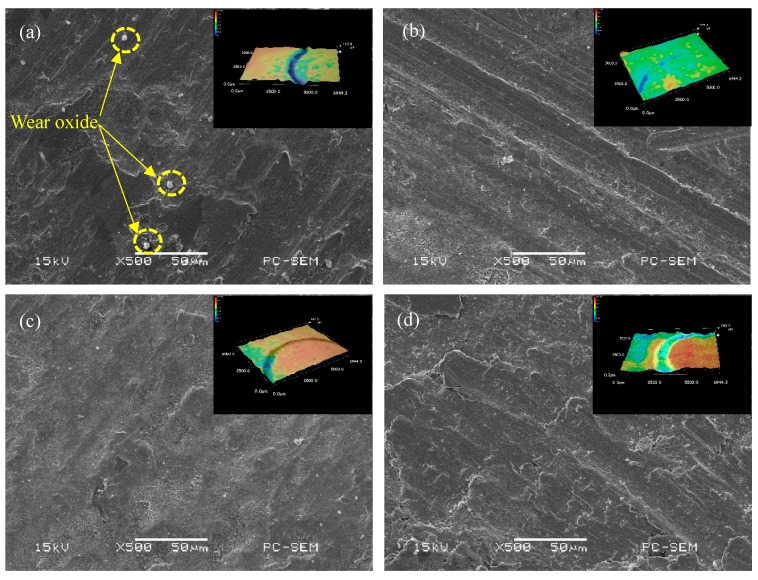
Wear micro-morphology and three-dimensional morphology of coatings with different CeO_2_ content: (**a**) 0.0% CeO_2_, (**b**) 1.0% CeO_2_, (**c**) 1.5% CeO_2_, and (**d**) 2.0% CeO_2_.

Table 2 shows the results of the wear parameters of the coating. When CeO_2_ is not added, the wear rate of the coating is 1.01 × 10^−5^ mm^3^·N^−1^·s^−1^. When CeO_2_ is added, the wear rate of the coating is obviously lower than that of the coating without CeO_2_, but it is not that the higher the CeO_2_ content is, the lower the wear rate of the coating is. When the CeO_2_ content is more than 1.5%, the wear rate of the coating decreases, and when it reaches 2.0%, the wear rate of the coating is close to that of the unadded rare earth coating. Figure 8 shows the worn morphology of the coating. When CeO_2_ is not added, white granular wear oxide appears on the worn surface, the worn surface is rough and the local damage is serious, and the wear mechanism is mainly micro-cutting and oxidation wear. When the content of CeO_2_ is 1.0%, the worn surface is flat and shallow ploughing appears, and the main wear mechanism is abrasive wear. When the content of CeO_2_ is 1.5%, there are pits and local damage on the worn surface and the wear mechanism is mainly micro-cutting. When the content of CeO_2_ is 2.0%, the worn surface is seriously damaged, and the main wear mechanism is micro-cutting and adhesive wear [25,26]. Based on the above results, it can be seen that rare earth elements have a certain effect on the wear resistance of the coating. The addition of appropriate amount of CeO_2_ can refine the microstructure, reduce the dendrite arm spacing and improve the toughness of the coating. When the content of CeO_2_ is more than 1%, the flowability of the molten pool decreases, which leads to the increase of the internal inclusions formed by cerium oxide and other components. In addition, in the process of solidification in the molten pool, deoxidation of CeO_2_ is reduced, so that the bubbles and inclusions are not easy to be discharged and inclusion in the coating increases, which leads to the decrease of plasticity and toughness and the wear resistance of the coating. 

Table 3 shows the impact test results of the impact specimen. It can be seen from the table that the impact toughness of the coating is 32.25 J/cm^2^ without the addition of CeO_2_. When the content of CeO_2_ is 1%, the impact toughness of the coating is the highest, which reaches 42.27 J/cm^2^. When the CeO_2_ content is further increased, the impact toughness of the coating decreases. When the CeO_2_ content reaches 2%, the impact toughness of the coating is almost the same as that of the coating without CeO_2_, which is maintained at 32 J/cm^2^. After laser cladding, the impact toughness of the substrate (28.5 J/cm^2^) can be increased up to 48%, and the toughness is obviously improved. Due to the characteristics of rapid solidification of laser cladding, the boundary and phase interface between dendrite and eutectic per unit volume will increase, and the increase of interface will hinder the movement of dislocations. Moreover, due to the laser thermal radiation effect, the high density dislocation martensite is formed in the heat shadow region, which is beneficial to improve the impact properties. On the other hand, due to the fine grain strengthening effect of rare earth CeO_2_, it can effectively prevent the growth of grains, hinder the movement of dislocations, increase the difficulty of crack propagation, and effectively improve the comprehensive mechanical properties of materials.

**Figure 9:**
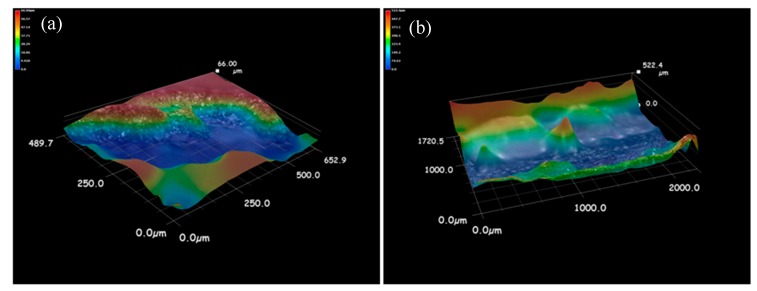

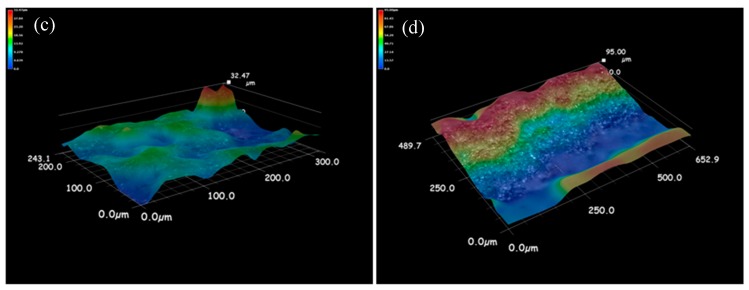
3D morphology of impact fracture surface with different CeO_2_ content: (**a**) 0.0% CeO_2_, (**b**) 1.0% CeO_2_, (**c**) 1.5% CeO_2_, and (**d**) 2.0% CeO_2_.

**Figure 10:**
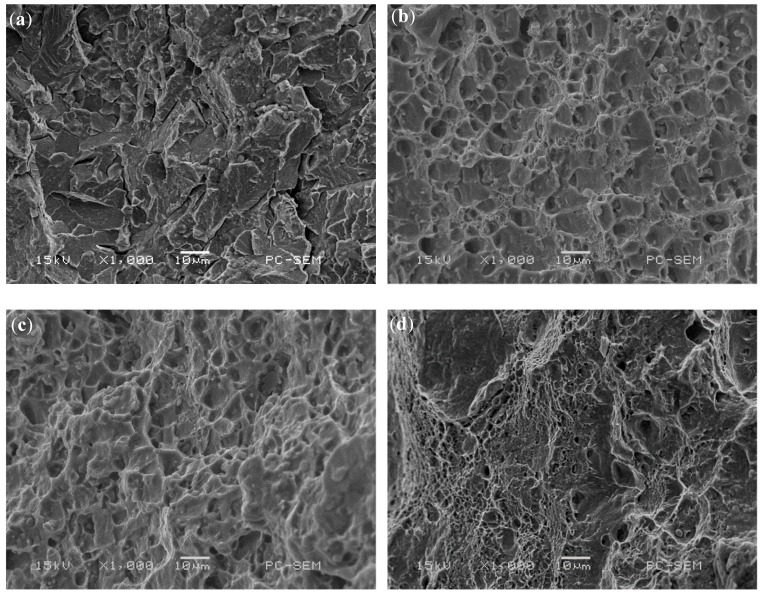
Impact fracture morphologies of Coatings with different CeO_2_ content: (**a**) 0.0% CeO_2_, (**b**) 1.0% CeO_2_, (**c**) 1.5% CeO_2_, and (**d**) 2.0% CeO_2_.

Figure 9 shows the 3D morphology of the fracture surface of the impact specimen. It can be seen that the fracture surface of the coating with rare earth is obviously uneven, and the specific surface area of the fracture is larger than that of the coating without rare earth. Because the impact fracture direction is perpendicular to the interface between the enhanced zone and the heat affected zone, when the crack propagation front enters the heat affected zone from the enhanced zone, the changes of hardness and toughness will make the crack propagation direction deflected or forked. This deflection and bifurcation increases the total area of the crack and increases the total energy absorbed by the crack propagation, which leads to the increase of the impact energy. Figure 10 shows the microscopic morphology of impact fracture. When CeO_2_ is not added, the fracture surface of the specimen is mainly composed of a small cleavage plane, which is connected by small and curved tear edges, and no dimples are found. At the same time, there are obvious river patterns and cleavage steps on the large cleavage plane, the fracture characteristics are quasi-cleavage fracture, and the surface has radial steps, which is the characteristic of brittle fracture [27]. When the CeO_2_ content is 1% and 1.5%, the fracture surface of the specimen is composed of dimples, and the plastic deformation mainly occurs in the process of crack propagation. In the same direction, the dimple of 1% CeO_2_ content sample is deeper and larger than that of 1.5% CeO_2_ content sample. This shows that the plastic deformation of 1% CeO_2_ content coating is larger than that of 1.5% CeO_2_ content coating under impact load, and the plastic deformation work consumed during crack propagation is larger than that of 1.5% CeO_2_ content coating. When the content of CeO_2_ is 2%, the fracture morphology of the sample is composed of a large number of dimples and cleavage planes, and the dimple band is surrounded by cleavage planes and tear edges, and the secondary crack is not obvious, which is a typical quasi-cleavage fracture morphology [28]. It can be seen that the fracture mechanism of impact specimen is the mixed fracture of dimple fracture and dissociative fracture. To sum up, the impact toughness of the coating with rare earth is better than that of the sample without rare earth.

**Figure 11:**
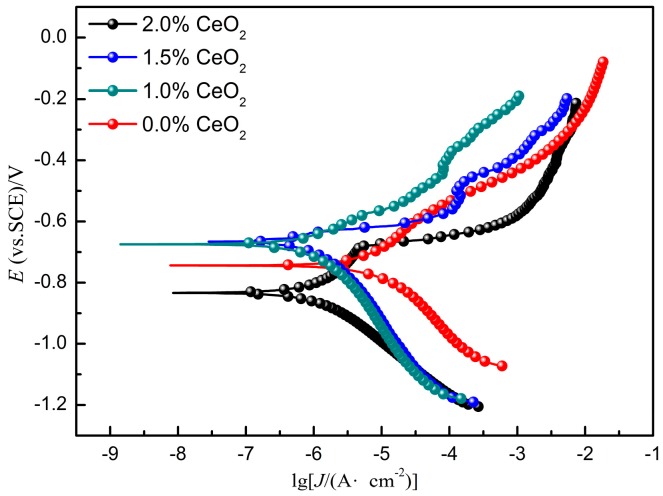
Polarization curves of coatings with different CeO_2_ content.

Figure 11 shows the potentiodynamic polarization curves of cladding coatings with different CeO_2_ content combined with the polarization curve fitting parameters shown in Table 4. Passivation occurred in all the four coatings. Without rare earth addition, the corrosion potential of the coating is −0.7348 V, the pitting potential is approximately −0.7 V, and the width of the passivation range is 0.3 V. When the rare earth content is 1%, the self-corrosion potential increases rapidly to −0.6 V, and the pitting potential also increases to −0.5 V. With the further increase of CeO_2_ content, the self-corrosion potential and pitting potential of the coating decreased. When the content of CeO_2_ reaches 2%, the self-corrosion potential and current density of the coating are lower than those of the coating without rare earth. There are two main reasons for this phenomenon. First, CeO_2_ can refine the crystal structure and improve the crystal composition segregation, so that the structure can be purified and the grain boundary corrosion rate can be delayed. Second, CeO_2_ can improve the content and distribution of corrosion resistance elements such as Ni and Ti, promote the corrosion resistance of the coating.

**Figure 12:**
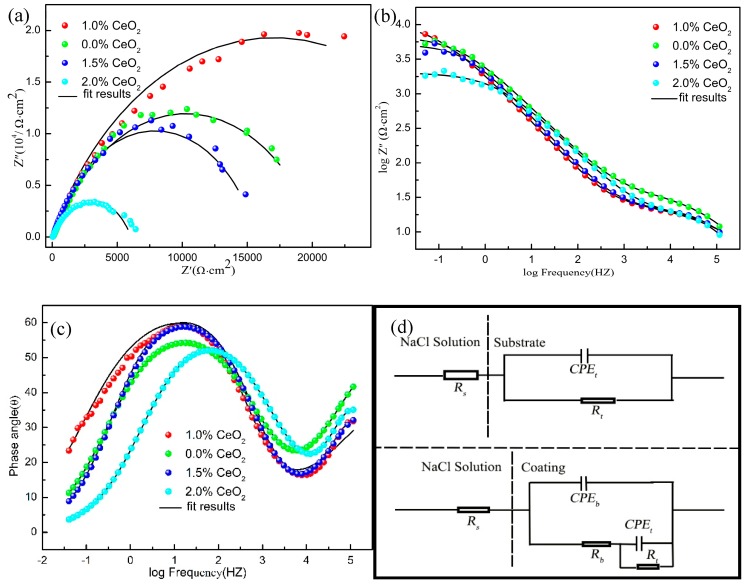
Impedance spectrum of coating in 3.5% NaCl solution: (**a**) Nyquist plot, (**b**,**c**) Bode plot, and (**d**) equivalent circuit.

In order to further study the electrochemical behavior of CeO_2_ on coatings, the impedance spectra of different coatings were measured, as shown in Figure 12. It can be seen that on the Nyquist diagram, the four kinds of coatings show capacitive arc at high frequency. The relationship between the radius of capacitance arc and the content of CeO_2_ is 2% < 1.5% < 0.0% < 1%. The larger the radius of arc resistance is, the better the corrosion resistance is. From the Bode diagram, we can see that all coatings correspond to two time constants at frequencies of 10 and 10,000. At low frequency, the impedance is higher when the content of CeO_2_ is 1.5%, 0.0%, and 1%. The interface reacted by capacitance arc resistance in low frequency region is corrosion product layer, and the interface reacted by capacitance arc resistance at high frequency is passivation film, because of the large phase angle, the passivation film is more compact [29]. When the content of CeO_2_ is 2.0%, the impedance in the low frequency region is low, and the interface reacted by the capacitance arc is caused by activation, indicating that the charge has been transferred, indicating the formation of pitting corrosion. Figure 12d shows the EIS equivalent circuit diagram. *R*_s_ represents the resistance of the corrosion medium, CPE represents the original of constant phase angle, *R*_b_ represents the resistance between the medium and the sample interface, and *R*_t_ represents the resistance on the coating surface dissolved by the corrosion medium. The corresponding equivalent circuit parameters are listed in Table 5, when the CeO_2_ content is 1.0%, the passivation film *R*_t_ of the coating is the largest, indicating that the corrosion resistance of the coating is the best.

## 4. Conclusions

(1) WC-reinforced Al-based composite coating with added CeO_2_ was prepared by laser cladding. The addition of CeO_2_ did not change the phase composition of the coating. Appropriate amount of CeO_2_ can enhance the flowability of the molten pool, refine the microstructure of the coating, and make the distribution of elements in the coating uniform. However, excessive CeO_2_ makes the diffusion of the molten pool difficult, and CeO_2_ will dissolve into the metal compounds, which makes the microstructure of the coating become thick again.
(2) Appropriate amount of rare earth CeO_2_ can significantly improve the hardness of the cladding coating. When the content of CeO_2_ is more than 1%, the fine grain strengthening effect decreases gradually, the dislocation movement increases, and the inclusions in the coating increase, resulting in the decrease of plasticity and impact toughness of the coating. Wear resistance is also reduced.
(3) The coating prepared with different CeO_2_ content has higher impedance and higher corrosion resistance than that of the substrate. When the content of CeO_2_ is 1%, the coating shows its best corrosion resistance.

## Figures and Tables

**Table 1:** Chemical composition of S420 steel (wt.%).

C	Si	Mn	P	Cr	S	Ni	Mo	Al	Ti	Nb	Cu	Fe
0.07	0.20	1.52	0.004	0.02	0.035	0.44	0.01	0.052	0.013	0.018	0.23	97.38

**Table 2:** Wear test results of coatings.

CeO_2_ Content (wt.%)	Wear Width (μm)	Wear Depth (μm)	Wear Area (mm^2^)	Wear Volume (mm^3^)	Wear Rate (mm^3^·N^−1^·s^−1^)
0.0	500.12	34.91	9.42	0.32	1.01 × 10^−5^
1.0	342.91	9.86	6.46	0.06	1.87 × 10^−6^
1.5	408.42	15.49	7.69	0.12	3.75 × 10^−6^
2.0	512.82	31.82	9.66	0.31	9.68 × 10^−6^

**Table 3:** Impact test data of different coatings.

CeO_2_ Contents (wt.%)	Impact Absorbing Energy *A*_k_ (J)	Fracture Surface Area S (cm^2^)	Impact Toughness *α*_k_ (J/cm^2^)
0.0	25.8	0.8	32.25
1.0	33.82	0.8	42.27
1.5	31.25	0.8	39.07
2.0	26.12	0.8	32.65

**Table 4:** Electrochemical data of coatings with different contents of CeO_2_.

CeO_2_ (wt.%)	*E_corr_* (V)	*i_corr_* (A/cm^2^)	*β_a_*/(mV)	*β_b_*/(mV)	Rp/(Ω·cm^2^)
0.0	−0.7348	3.7721 × 10^−6^	302.23	201.76	2.9228 × 10^−5^
1.0	−0.6661	8.3794 × 10^−7^	150.73	122.74	3.9934 × 10^−6^
1.5	−0.6745	7.7306 × 10^−7^	102.82	189.78	3.3773 × 10^−6^
2.0	−0.8335	1.7053 × 10^−6^	208.49	227.39	2.2758 × 10^−5^

**Table 5:** EIS data of substrate and coatings with different contents of CeO_2_.

CeO_2_ (wt.%)	R_s_ (Ω·cm^2^)	Q_b_ (Ω^−1^·s^−n^·cm^−2^)	N_b_	R_b_ (Ω·cm^2^)	Q_t_ (Ω^−1^·s^−n^·cm^−2^)	N_t_	R_t_ (Ω·cm^2^)
0.0	0.33	7.713 × 10^−5^	0.7024	26.55	8.277 × 10^−5^	0.7673	11,970
1.0	3.377	3.229 × 10^−6^	0.7649	18.66	9.661 × 10^−5^	0.7374	36,796
1.5	4.62	3.515 × 10^−6^	0.9551	13.66	9.670 × 10^−5^	0.673	35,230
2.0	0.3571	8.11 × 10^−6^	0.6638	36.25	8.824 × 10^−5^	0.675	20,000
